# Systematic review and meta-analysis of totally laparoscopic *versus* laparoscopic assisted distal gastrectomy for gastric cancer

**DOI:** 10.1186/s12957-015-0532-7

**Published:** 2015-03-21

**Authors:** Yi-Xin Zhang, Ying-Jie Wu, Guo-Wen Lu, Min-Ming Xia

**Affiliations:** Department of Surgical Oncology, Yinzhou Affiliated Hospital of Medical College of Ningbo University, 251 Baizhang Road, Ningbo, Zhejiang 315040 People’s Republic of China

**Keywords:** Gastric cancer, Laparoscopic gastrectomy, Intracorporeal anastomosis, Meta-analysis

## Abstract

**Background:**

Totally laparoscopic distal gastrectomy (TLDG) has been developed in the hope of improving surgical quality and overcoming the limitations of conventional laparoscopic assisted distal gastrectomy (LADG) for gastric cancer. The aim of this study was to determine the extent of evidence in support of these ideals.

**Methods:**

A systematic review of the two operation types (LADG and TLDG) was carried out to evaluate short-term outcomes including duration of operation, retrieved lymph nodes, estimated blood loss, resection margin status, technical postoperative complications, and hospital stay.

**Results:**

Twelve non-randomized observational clinical studies involving 2,255 patients satisfied the eligibility criteria. Operative time was not statistically different between groups (*P* > 0.05). The number of retrieved lymph nodes and the resection margin length in TLDG were comparable with those in LADG. Estimated blood loss was significantly less in TLDG than that in LAG (*P* < 0.01). Compared to LADG, TLDG also involved lesser postoperative hospital stay (*P* < 0.01) and earlier time to soft diet intake (*P* < 0.05). Time to flatus and postoperative complications were similar for those two operative approaches.

**Conclusions:**

TLDG may be a technically safe, feasible, and favorable approach in terms of better cosmesis, less blood loss, and faster recovery compared with LADG.

## Background

Since it was first reported in 1994 [1], laparoscopy-assisted distal gastrectomy (LADG) for gastric cancer has undergone rapid development and gained popularity in the past 20 years. Compared to traditional open gastrectomy, most studies have reported that LADG can achieve better cosmesis, shorter hospital stay, faster postoperative recovery, and better postoperative quality of life [2-5]. Some studies reported that patients who receive laparoscopic gastrectomy (LG) have similar clinical benefits in the long-term as those who receive laparotomy [6,7].

The most popular version of LG is laparoscopic-assisted distal gastrectomy (LADG) for lower gastric carcinoma, wherein the lymph node dissection is completed under the laparoscope. An epigastrium auxiliary incision is then made to facilitate the excision of the specimen and the reconstruction of the digestive tract. Another version is the totally laparoscopic gastrectomy (TLDG) also for lower gastric carcinoma, which is characterized by an intracorporeal anastomosis without auxiliary incision and no touching of the tumor; it is considered ‘incisionless’, with the exception of the trocar wounds [8]. However, less studies have focused on the feasibility and safety of TLDG, which we consider as a laparoscopic approach with intracorporeal anastomosis, given the safety concerns associated with laparoscopic reconstruction of the gastrointestinal tract. Nevertheless, the anastomosis procedure, which distinguishes assisted from totally laparoscopic surgery, could affect the short-term outcomes of this type of surgery. Thus, it might prove interesting to compare the outcomes of TLDG with LADG. And, we performed this meta-analysis to clarify the feasibility and safety of TLDG and assess the relative merits of TLDG comparing with LADG.

## Methods

### Search strategy

Systematic searches of PubMed, Cochrane Library, and Web of Science were performed to identify articles published up to February 2015 that compared LADG and TLDG. The search terms ‘gastric adenocarcinoma’, ‘gastric cancer’, ‘laparoscopic’, ‘laparoscopy’, ‘gastrectomy’, ‘completely’, ‘entirely’, ‘totally’, ‘intracorporeal,’ and ‘endocorporeal’ were utilized. All references of retrieved articles were reviewed to identify all the potential studies. The language of the publications was confined to English.

### Eligibility criteria

All clinical studies should meet the following criteria for the meta-analysis: (1) published in English with data comparing LADG and TLDG; (2) clear case selection criteria, containing at least the following information: the number of cases, surgical methods, and perioperative data; and (3) if there was overlap between authors or centers, the higher quality or more recent literature was selected. The papers containing any of the following were excluded: (1) laparoscopic hand-assisted or robot-assisted gastrectomy; (2) total gastrectomy or proximal gastrectomy; (3) non-gastric carcinoma cases; and (4) studies in which <2 of the indices under study were reported, or it was difficult to calculate these from the results.

### Data extraction and quality assessment

Two authors independently extracted the data using a unified datasheet, and controversial issues were decided by discussion. The extracted data included: author, study period, geographical region, number of patients, operation time, blood loss, number of retrieved lymph nodes, proximal and distal margin distance, analgesic use, time to flatus, time to oral intake, length of hospital stay, and morbidity, mortality and anastomotic-related complications, which were classified as anastomotic leakage, stenosis, and hemorrhage. The quality of the observational clinical studies was assessed using the Newcastle-Ottawa Quality Assessment Scale (NOS). This scale varies from 0 to 9 stars: studies with a score equal to or higher than 6 were considered methodologically sound.

### Statistical analysis

Continuous variables were assessed using weighted mean difference (WMD), and dichotomous variables were analyzed using the risk ratio (RR). Statistical heterogeneity, which indicated between-study variance, was evaluated according to the Higgins *I*^2^ statistic [9]. To account for clinical heterogeneity, which refers to diversity relevant to clinical situations, we used the random-effects model based on DerSimonian and Laird’s method. Potential publication bias was determined by conducting an informal visual inspection of funnel plots based on the complications. Data analyses were performed using Review Manager Version 5.1 (RevMan 5.1) software downloaded from the Cochrane Library. *P* < 0.05 was considered statistically significant.

## Results

### Studies selected

**Figure 1:**
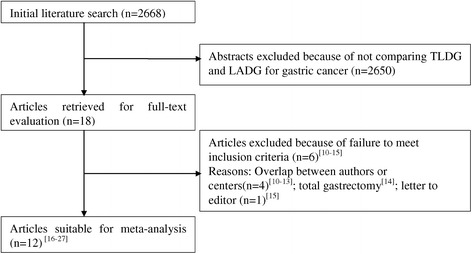
**Flow chart of literature search strategies.** LADG, laparoscopic assisted distal gastrectomy; TLDG, totally laparoscopic distal gastrectomy [10-27].

The initial search strategy retrieved 2,668 publications in English. After the titles and abstracts were reviewed, papers without a comparison of LADG and TLDG were excluded, which left 18 comparative studies, 6 [10-15] of which did not meet the inclusion criteria and were excluded. This left a total of 12 observational studies [16-27], all of which were accessible in full-text format. A flow chart of the search strategies, which includes the reasons for excluding studies, is illustrated in Figure 1.

### Study characteristics and quality

A total of 2,255 patients were included in the analysis with 1,228 undergoing LADG (54.5%) and 1,027 undergoing TLDG (45.5%). All studies had Asian data from Japan, Korea, and China (three from Japan, seven from Korea, two from China). In general, the quality of the included studies was satisfactory. According to the NOS, 1 out of the 12 observational studies got 9 stars, 1 article got 8 stars, 7 articles got 7 stars, and the remaining 3 got 6 stars. Table 1 presents the characteristics of the included studies, whereas Table 2 presents the quality assessment based on the NOS.

**Table 1:** **Summary of studies included in the meta-analysis**

**Author**	**Nation**	**Year**	**Study period**	**Sample Size**		**Level of Lymphadenectomy**	**Reconstruction**
				**LADG**	**TLDG**		
Song [16]	Korea	2008	2005 to 2006	20	20	D1 + β, D2	B-I, B-II, R-Y
Ikeda [17]	Japan	2009	2005 to 2007	24	56	D1 + β, D2	B-I, R-Y
Kinoshita [18]	Japan	2011	2007 to 2009	41	42	D1 + α/β, D2	B-I
Kim MG [19]	Korea	2011	2009 to 2010	328	239	D2	B-I
Lee [20]	Korea	2012	2004 to 2011	269	130	D1 + α/β, D2	B-II
Kim DG [21]	Korea	2013	2009 to 2012	106	60	D1 + β, D2	B-I
Kim HG [22]	Korea	2013	2005 to 2012	136	111	D1 + β, D2	B-I, B-II
Choi [23]	Korea	2013	2007 to 2012	35	37	D1 + α/β, D2	B-I, B-II, R-Y
Chen [24]	China	2014	2004 to 2013	93	147	D2	B-I, B-II
Zhang [25]	China	2014	2012 to 2013	25	11	D2	B-II
Han [26]	Korea	2014	2005 to 2013	77	134	D1 + α/β, D2	B-I, B-II, R-Y
Kanaji [27]	Japan	2014	2010 to 2012	74	40	D1 + α/β, D2	B-I, B-II, R-Y

B-I, Billroth-I; B-II, Billroth-II; R-Y: Roux-en-Y.

**Table 2:** **Quality assessment based on the NOS for observational studies**

**Author**	**Selection (out of 4)**				**Comparability (out of 2)**	**Outcomes (out of 3)**			**Total (out of 9)**
	**Representativeness of exposed cohort**	**Selection of non-exposed cohort**	**Ascertainment of exposure**	**Outcome not present at the start of the study**		**Assessment of outcomes**	**Length of follow-up**	**Adequacy of follow-up**	
Song	*	*	*	*	**	*			7
Ikeda	*	*	*	*	**	*			7
Kinoshita	*	*	*	*	**	*		*	8
Kim MG	*	*	*	*	*	*			6
Lee	*	*	*	*	**	*			7
Kim DG	*	*	*	*	*	*		*	7
Kim HG	*	*	*	*	*	*			6
Choi	*	*	*	*	**	*			7
Chen	*	*	*	*	**	*	*	*	9
Zhang	*	*	*	*	**	*			7
Han	*	*	*	*	*	*			6
Kanaji	*	*	*	*	**	*			7

The NOS scale varies from 0 to 9 stars: studies with a score equal to or higher than 6 were considered methodologically sound. According to the NOS, 1 out of the 12 observational studies got 9 stars, 1 article got 8 stars, 7 articles got 7 stars, and the remaining 3 got 6 stars.

### Intraoperative effects

**Figure 2:**
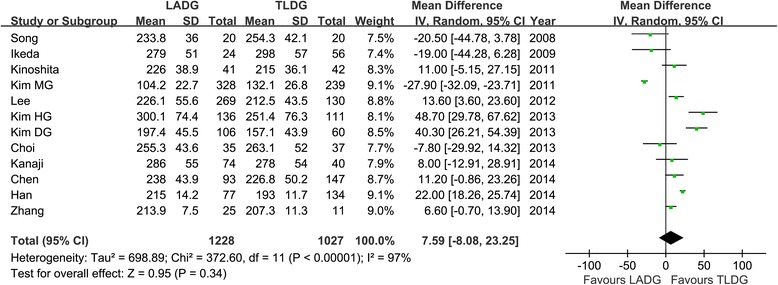
**Meta-analysis of the pooled data: operation time.** CI, confidence interval; LADG, laparoscopic assisted distal gastrectomy; SD, standard deviation; TLDG, totally laparoscopic distal gastrectomy.

**Figure 3:**
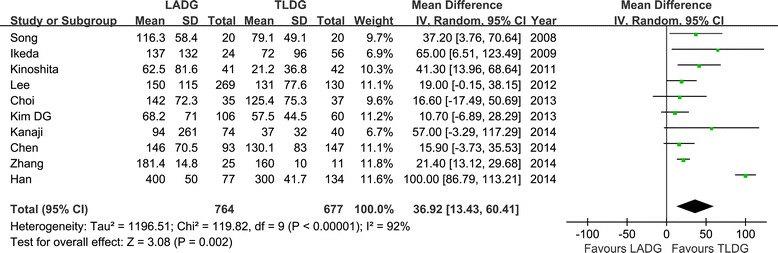
**Meta-analysis of the pooled data: intraoperative blood loss.** CI, confidence interval; LADG, laparoscopic assisted distal gastrectomy; SD, standard deviation; TLDG, totally laparoscopic distal gastrectomy.

**Figure 4:**
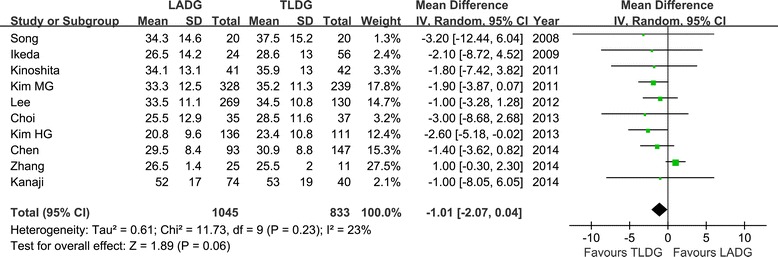
**Meta-analysis of the pooled data: retrieved lymph nodes.** CI, confidence interval; LADG, laparoscopic assisted distal gastrectomy; SD, standard deviation; TLDG, totally laparoscopic distal gastrectomy.

All 12 studies reported operation time [16-27]. The present analysis showed no statistically significant difference in the operation time of the two groups (WMD = 7.59 min; 95% CI, −8.08 to 23.25; *P* = 0.34) (Figure 2). Ten studies reported blood loss [16-18,20,21,23-27]. Intraoperative blood loss was significantly lower in the TLDG compared with the LADG group (WMD = 36.92 ml; 95% CI, 13.43 to 60.41 ml; *P* < 0.01) (Figure 3). No statistical difference was found between the two groups in terms of the number of harvested lymph nodes (WMD = −1.01; 95% CI, −2.07 to 0.07; *P* = 0.06) (Figure 4). The length of the proximal resection margin was similar in both groups (WMD = −0.48 cm; 95% CI, −1.28 to 0.31 cm; *P* = 0.23). However, the distal margin distance of TLDG was longer than that of the LADG with a marginal difference (WMD = −0.51 cm; 95% CI, −1.06 to 0.05 cm; *P* = 0.07). All intraoperative effect outcomes are summarized in Table 3.

**Table 3:** **Pooled short-term outcomes of meta-analysis**

**Outcomes**	**Number of studies**	**Sample size**		**Heterogeneity (** ***P, I*** **2)**	**Overall effect size**	**95% CI of overall effect**	***P*** **value**
		**LADG**	**TLDG**				
Operation time (min)	12	1,228	1,027	<0.01, 97%	WMD = 7.59	−8.08 to 23.25	0.34
Blood loss (ml)	10	764	677	<0.01, 92%	WMD = 36.92	13.43 to 60.41	<0.01
Retrieved lymph nodes	10	1,045	833	0.23, 23%	WMD = −1.01	−2.07 to 0.07	0.06
Proximal margin (cm)	8	982	874	<0.01, 96%	WMD = −0.48	−1.28 to 0.31	0.23
Distal margin (cm)	5	846	647	<0.01, 74%	WMD = −0.51	−1.06 to 0.05	0.07
Analgesics use (times)	5	764	491	<0.01, 84%	WMD = 0.52	−0.17 to 1.21	0.14
Time to first flatus (days)	10	882	763	<0.01, 96%	WMD = 0.23	−0.13 to 0.59	0.21
Time to liquid diet (days)	6	292	333	<0.01, 92%	WMD = 0.30	−0.15 to 0.75	0.19
Time to soft diet (days)	5	571	522	<0.01, 81%	WMD = 0.60	0.04 to 1.17	0.04
Hospital stay (days)	12	1228	1027	<0.01, 80%	WMD = 0.68	0.17 to 1.18	<0.01
Overall complications	12	1228	1027	0.41, 3%	RR = 0.97	0.75 to 1.27	0.85

RR, risk ratio; WMD, weighted mean difference.

### Postoperative outcome

**Figure 5:**
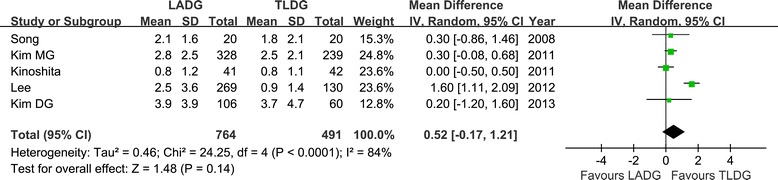
**Meta-analysis of the pooled data: analgesics use.** CI, confidence interval; LADG, laparoscopic assisted distal gastrectomy; SD, standard deviation; TLDG, totally laparoscopic distal gastrectomy.

Postoperative pain was evaluated by the times of analgesics use [16,18-21]. Although, the mean times of analgesics use was less in the TLDG group, it failed to reach statistically significant (WMD = 0.52; 95% CI, −0.17 to 1.21; *P* = 0.10) (Figure 5).

**Figure 6:**
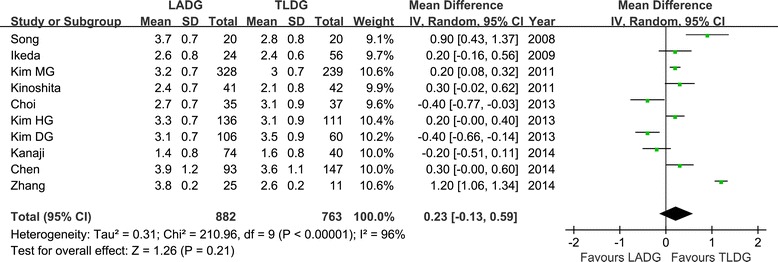
**Meta-analysis of the pooled data: time to first flatus.** CI, confidence interval; LADG, laparoscopic assisted distal gastrectomy; SD, standard deviation; TLDG, totally laparoscopic distal gastrectomy.

**Figure 7:**
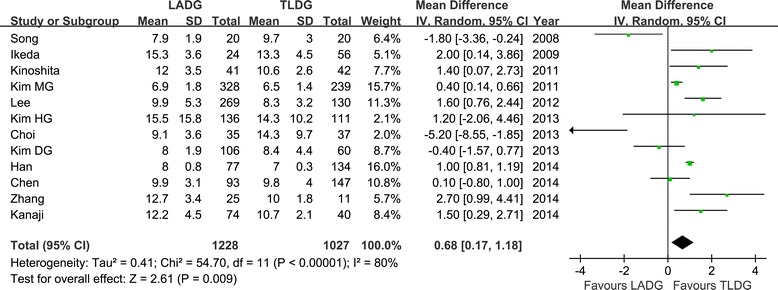
**Meta-analysis of the pooled data: postoperative hospital stay.** CI, confidence interval; LADG, laparoscopic assisted distal gastrectomy; SD, standard deviation; TLDG, totally laparoscopic distal gastrectomy.

Flatus is one of the outcome measures for evaluating postoperative recovery of gastrointestinal functions [16-19,21-25,27]. No significant difference between the two groups was observed regarding the time to first flatus (WMD = 0.23 day; 95% CI −0.13 to 0.59 day; *P* = 0.21), as was the time to restart liquid diet (WMD = 0.30 day; 95% CI, −0.15 to 0.75; *P* = 0.19) (Figure 6). However, patients in the TLDG group were able to resume soft diet earlier (WMD = 0.60 day; 95% CI, 0.04 to 1.17; *P* = 0.04) after surgery. Moreover, the mean postoperative hospital stay was 0.68 day shorter for TLDG patients with a significant difference (WMD = 0.68 day; 95% CI, 0.17 to 1.18; *P* < 0.01) (Figure 7).

Five studies reported inflammatory response index such as white blood cell (WBC) count and C-reactive protein (CRP) [16-18,20,21]. The outcomes were divergent, and only two studies reported a significantly lower CRP count for TLDG compared with LADG on postoperative day 7 [17,18].

**Figure 8:**
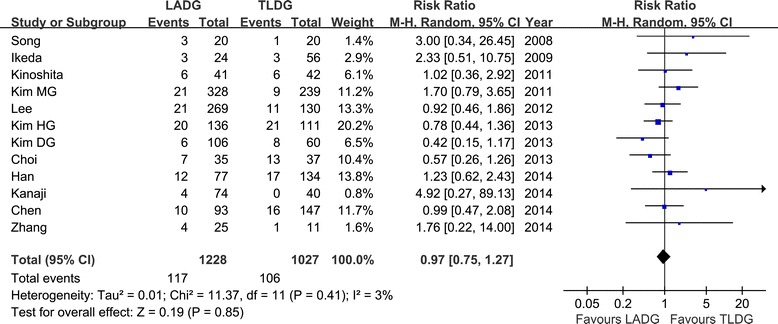
**Meta-analysis of the pooled data: overall complications.** CI, confidence interval; LADG, laparoscopic assisted distal gastrectomy; TLDG, totally laparoscopic distal gastrectomy.

**Figure 9:**
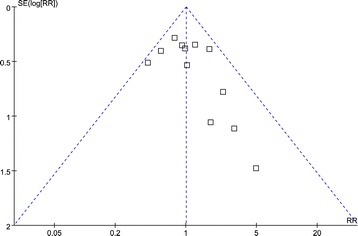
**Funnel plots of the overall postoperative complications.** RR, risk ratio; SE, standard error.

Morbidity was described in all 12 studies [16-27], and there was no significant difference in postoperative morbidity (RR = 0.97, 95% CI, 0.75 to 1.27, *P* = 0.85) (Figure 8). Visual inspection of the funnel plot revealed symmetry, indicating no serious publication bias (Figure 9). After further analysis, anastomotic-related complications, which were classified as anastomotic leakage, stenosis, and hemorrhage, were also similar between the two groups (RR = 0.86, 95% CI, 0.46 to 1.63, *P* = 0.65), as was the rate of abdominal abscess or fluid collection (RR = 1.08, 95% CI, 0.51 to 2.29, *P* = 0.84). The specific postoperative complications included in the studies are summarized in Table 4.

**Table 4:** **Systematic review of postoperative complications**

**Author**	**Group**	**Number**	**Total event**	**Specified complications**
Song	LADG	20	3	Wound infection × 1,ileus × 1,abdominal abscess × 1
	TLDG	20	1	Abdominal bleeding × 1
Ikeda	LADG	24	3	Wound infection × 1, atelectasis × 2
	TLDG	56	3	Wound infection × 2,pancreatic leakage × 1
Kinoshita	LADG	41	6	Wound infection × 2,abdominal abscess × 1, anastomotic hemorrhage × 1, anastomotic stricture × 1, pancreatitis × 1
	TLDG	42	6	Wound infection × 2,abdominal abscess × 2, pancreatitis × 1, cholecystitis × 1
Kim MG	LADG	328	21	Wound complications × 11, anastomosis leakage × 2, anastomosis stenosis × 1, anastomosis bleeding × 2, abdominal abscess × 4, abdominal bleeding × 1
	TLDG	239	9	Wound complications × 2, anastomosis leakage × 1, anastomosis bleeding × 1,abdominal abscess × 3, abdominal bleeding × 1, paralytic ileus × 1
Lee	LADG	269	21	Duodenal stump leakage × 10, anastomosis leakage × 3, abdominal bleeding × 2, afferent loop obstruction × 1, gastric stasis × 5
	TLDG	130	11	Duodenal stump leakage × 3, anastomosis leakage × 1, abdominal bleeding × 4, afferent loop obstruction × 1, gastric stasis × 2
Kim DG	LADG	106	6	Anastomosis leakage × 1,fluid collection × 4, respiratory infection × 1
	TLDG	60	8	Anastomosis leakage × 1,fluid collection × 2, respiratory infection × 1, wound seroma × 1, delayed gastric emptying × 2, trocar site hernia × 1
Kim HG	LADG	136	20	Anastomotic leakage × 6, abdominal abscess × 2, wound problem × 4, abdominal bleeding × 2, pancreatitis × 1, lung complication × 2, hepatic complication × 1, outlet obstruction × 1,
	TLDG	111	21	Anastomotic leakage × 3, abdominal abscess × 2, wound problem × 2, abdominal bleeding × 4, luminal bleeding × 2, ileus × 2, pancreatitis × 3, hepatic complication × 3, heart complication × 2, stasis × 2
Choi	LADG	35	7	Wound infection × 1, stasis × 2, leakage × 1, pulmonary complication × 2, renal complication × 1, cerebral infarction × 1
	TLDG	37	13	Wound infection × 1, abdominal abscess × 1, leakage × 4, abdominal bleeding × 1, pancreatitis × 2, pulmonary complication × 2, hepatic complication × 1,
Chen	LADG	93	10	Anastomosis leakage × 1, hemorrhage × 1, abdominal abscess × 3, pulmonary infection × 2,delayed gastric emptying × 2, lymphorrhea × 1
	TLDG	147	16	Anastomosis leakage × 1, hemorrhage × 2, abdominal abscess × 2, pulmonary infection × 3,delayed gastric emptying × 4, lymphorrhea × 2, ileus × 1, pancreatic fistula × 1
Zhang	LADG	25	4	Wound infection × 2,ileus × 2
	TLDG	11	1	Duodenal stump leakage × 1
Kanaji	LADG	74	4	Delayed gastric emptying × 1, wound infection × 2, acute pulmonary edema × 1
	TLDG	40	0	

## Discussion

Currently, the safety and therapeutic effect of LADG has been preliminarily confirmed [28]. However, extracorporeal anastomosis is conducted in a limited working space with restricted vision, thus making it a difficult procedure, especially on obese patients [29]. Extension of the laparotomy is often necessary to obtain a better view for secure anastomosis following LADG on obese patients. TLDG was introduced in the hope of overcoming the difficulty of reconstruction, especially on obese patients. Lopez *et al*. [30] has reported TLDG surgery as early as in 1996, but this procedure was not popularized and developed in a long period of time due to the great difficulty in laparoscopic digestive tract reconstruction and concerns about the anastomotic security. In recent years, the development of laparoscopic instruments and the continuous accumulation of surgical experience contribute to the increasing maturity of laparoscopic gastrointestinal anastomosis technique. Especially, the emergence of delta-shaped method makes the laparoscopic Billroth I gastroduodenostomy possible, which greatly promotes the development of TLDG. A few reports have described the benefits of intracorporeal anastomosis, such as small wound size and early bowel recovery [31]. Regardless of the benefits, there was some fear that introducing TLDG would result in longer operation times, a higher incidence of operative complications, and more conversions to open laparotomy than LADG, especially during the introductory phase of TLDG. Given the lack of support from large-scale randomized controlled studies (RCTs), the security and mini-invasive therapeutic value of TLDG surgery are still controversial. Therefore, this research conducts a comprehensive analysis on the existing relevant data of TLDG-LADG comparative studies using meta-analysis to provide a relatively objective evaluation on TLG surgery.

Given the difficulty in laparoscopic digestive tract reconstruction, researchers worry that TLDG may lead to prolonged operative time. However, results of this study showed that the total operation time of the TLDG group was not longer than the LADG group. The study by Lee *et al*. [20] and Han *et al*. [26] even showed a shorter anastomosis time in the TLDG group than that in the LADG group. This may mainly result from the fact that the current laparoscopic Billroth I and Billroth II anastomoses are generally completed with the help of laparoscopic stapler instead of the laparoscopic suturing technique. Previous studies have reported that at least 20 to 40 cases are needed to stabilize the surgical procedure for TLDG and to overcome the initial learning curve even for surgeons with sufficient experience in laparoscopic gastrectomy [22,32]. TLDG could be introduced safely by surgical members who had experience with LADG. However, TLDG requires more skill with laparoscopic techniques than LADG, and it is necessary that a surgeon be well trained when beginning to perform TLDG. The most representative methods for distal gastrectomy are a delta-shaped anastomosis to perform a Billroth I gastroduodenostomy and a linear stapler method to perform a side-to-side Billroth II gastrojejunostomy [33,34]. In addition, TLDG avoids cutting and suturing of the small incision in epigastrium, thus leading to a shorter operation time. The intraoperative blood loss in the TLDG group is significantly lower than that in the LADG group. There are two reasons: increased bleeding resulted from auxiliary incision and excessive stretch for the remnant stomach may injure surrounding tissues and cause bleeding. However, this result should be interpreted prudently because the heterogeneity between studies was high and no details were reported in any studies pooled regarding the methods of estimating blood loss.

In terms of postoperative recovery measurements, the TLDG group was associated with earlier soft diet intake and postoperative hospital stay. Du *et al*. reported a significant less days of analgesics use in the TLDG group than the LADG group [35]. The TLDG group showed shorter post-hospital stay than the LADG group, and there were no differences in operative complication, WBC count, and level of serum CRP. TLDG has been shown to lead to earlier recovery of bowel function than with LADG and open resections. Small wound size, no incidence of operative complication, and earlier bowel function recovery appeared to be associated with shorter post-hospital stay of the TLDG group. There is a possibility that TLDG is a less invasive procedure than LADG.

Our study showed that there was no significant difference in the overall postoperative complications between the TLDG group and the LADG group. The results of in-depth analysis on anastomotic complications closely associated with digestive tract reconstruction (anastomotic leakage, anastomotic stenosis, and anastomotic bleeding) also showed no significant difference between these two groups. Some researches show that TLDG is not inferior to LADG in terms of the overall safety and the anastomotic-related safety [36,37]. In addition, some scholars believe that TLDG may increase the chance of abdominal infection due to its requirement to temporarily open the stomach cavity under laparoscopy [35]. However, this study also found no significant difference in abdominal infection between two groups. Adequate gastrointestinal decompression before opening the stomach cavity and local peritoneal washing after completing anastomosis could decrease abdominal infection [33].

Oncological outcome is a critical measure of success in laparoscopic surgery for malignant tumors. The number of the retrieved lymph nodes and surgical resection margin are the major indicators of oncological surgical quality. The present meta-analysis showed that the number of retrieved lymph nodes with TLDG was more than that for LADG with a marginal difference (*P* = 0.06). Causing lymphadenectomy in both approaches theoretically were similar, we inferred that most surgeons introduced TLDG when they had been experienced with LADG and proficient in lymphadenectomy. Besides, the asymmetric distribution of tumor classification or extent of lymphadenectomy makes comparison of harvested lymph nodes inherently flawed and at a high risk for confounding factors. Besides, our meta-analysis demonstrated a reduced distal margin in the LADG group compared with the TLDG group also with a marginal difference (*P* = 0.07). We argued that such a result may relate to the nature of LADG where the specimen is resected and reconstruction is performed through a mini-laparotomy, and it is difficult to pull the distal stomach using a narrow incision, which may influence the distance of the distal margin. TLDG avoids such difficulties, and a longer distal margin may be expected.

Jun *et al.* had conducted a meta-analysis previously and demonstrated that TLDG was associated with reduced blood loss and complications compared with LADG [38]. However, several studies pooled in Jun’s meta-analysis were small sample size and the amount of pooled studies was limited, which may influence the meta-analysis result severely. Besides, since the study by Jun *et al.* was published, several clinical observational studies have become available. The larger the number of patients in a meta-analysis, the greater its power to detect a possible treatment effect. Therefore, our comprehensive meta-analysis will contribute to a more systematic and objective evaluation for the safety and cancer treatment of TLDG.

Our present study has several limitations. First, observation bias: due to the varied measurement methods used by different authors, significantly different results were almost inevitable in the non-RCT or non-blind RCT studies. Second, publication bias: some gray literatures which contained negative results were difficult to obtain because most authors tended to show positive results. Third, grouping bias: notwithstanding the literatures dealing with significantly different diseases and surgical methods have been excluded in this study, in practice, patients should be grouped inevitably according to the disease condition and surgeons’ choices.

## Conclusions

The available clinical evidence implies that TLDG is a safe, feasible approach for patients with gastric cancer. The results of TLDG were favorable in terms of better cosmesis, less blood loss, and faster recovery. However, more methodologically high-quality comparative studies are required to adequately evaluate the status of TLDG.

## Abbreviations

CI: Confidence intervals
LADG: Laparoscopic assisted distal gastrectomy
LG: Laparoscopic gastrectomy
NOS: Newcastle-Ottawa quality assessment scale
OG: Open gastrectomy
RCT: Randomized controlled trial
RR: Risk ratio
SD: Standard deviation
TLDG: Totally laparoscopic distal gastrectomy
WMD: Weighted mean differences
